# Upper Extremity Functional Evaluation by Fugl-Meyer Assessment Scoring Using Depth-Sensing Camera in Hemiplegic Stroke Patients

**DOI:** 10.1371/journal.pone.0158640

**Published:** 2016-07-01

**Authors:** Won-Seok Kim, Sungmin Cho, Dongyoub Baek, Hyunwoo Bang, Nam-Jong Paik

**Affiliations:** 1 Department of Rehabilitation Medicine, Seoul National University College of Medicine, Seoul National University Bundang Hospital, Seongnam-si, Gyeonggi-do, South Korea; 2 Department of Mechanical and Aerospace Engineering, Seoul National University, Seoul, South Korea; INSERM U894, FRANCE

## Abstract

Virtual home-based rehabilitation is an emerging area in stroke rehabilitation. Functional assessment tools are essential to monitor recovery and provide current function-based rehabilitation. We developed the Fugl-Meyer Assessment (FMA) tool using Kinect (Microsoft, USA) and validated it for hemiplegic stroke patients. Forty-one patients with hemiplegic stroke were enrolled. Thirteen of 33 items were selected for upper extremity motor FMA. One occupational therapist assessed the motor FMA while recording upper extremity motion with Kinect. FMA score was calculated using principal component analysis and artificial neural network learning from the saved motion data. The degree of jerky motion was also transformed to jerky scores. Prediction accuracy for each of the 13 items and correlations between real FMA scores and scores using Kinect were analyzed. Prediction accuracies ranged from 65% to 87% in each item and exceeded 70% for 9 items. Correlations were high for the summed score for the 13 items between real FMA scores and scores obtained using Kinect (Pearson’s correlation coefficient = 0.873, *P*<0.0001) and those between total upper extremity scores (66 in full score) and scores using Kinect (26 in full score) (Pearson’s correlation coefficient = 0.799, *P*<0.0001). Log transformed jerky scores were significantly higher in the hemiplegic side (1.81 ± 0.76) compared to non-hemiplegic side (1.21 ± 0.43) and showed significant negative correlations with Brunnstrom stage (3 to 6; Spearman correlation coefficient = -0.387, *P* = 0.046). FMA using Kinect is a valid way to assess upper extremity function and can provide additional results for movement quality in stroke patients. This may be useful in the setting of unsupervised home-based rehabilitation.

## Introduction

Stroke is a leading cause of disabilities worldwide[1] and hemiplegia is the most common impairment after stroke, [2] resulting in upper extremity (UE) dysfunction. UE impairment is associated with limitation of activities and worse health-related quality of life.[3] Because recovery of UE impairment is marked in the first 6 to 12 months after stroke onset and can continue slowly up to one year,[4, 5] optimal rehabilitation is recommended for maximal recovery during this period, even after home discharge. However, only about 30% of stroke survivors in the United States receive outpatient stroke rehabilitation,[6] which is lower than the expected percentage considering the clinical practice guideline. This may be associated with barriers including costs, travel and limited use of public transportation due to disabilities.[6] Furthermore, adequate rehabilitation facilities are limited in developing countries and use of outpatient rehabilitation facilities are likely very low.[7]

A home-based virtual rehabilitation system could be a useful alternative for conventional rehabilitation to overcome barriers for outpatient rehabilitation in stroke patients, considering its low cost and greater accessibility.[7] Among many components in home-based rehabilitation systems, objective functional assessment is important to plan current function-based rehabilitation and monitor recovery as well as to motivate patients. Tele-based assessments by therapists using video are possible but may necessitate scheduling an appointment with the therapist and would involve additional cost. Some scores during virtual gaming can be used for assessment, but these are not intuitive, are typically not familiar to therapists.[8, 9]

The Fugl-Meyer Assessment (FMA) is a comprehensive measurement tool for motor function after stroke. FMA is valid, reliable and responsive to change.[10] Among the five FMA domains, the motor domain is most widely used and has the primary value of monitoring motor recovery after stroke. Most items in the UE motor domain are based on patient motion, although reflex or resistance has to be measured in a few items.[11] A depth-sensing camera, such as Kinect (Microsoft, USA), can detect joint movement three-dimensionally[12] and may be used to predict the FMA score. In one previous study, scores calculated from motion data captured by Kinect correlated well with the motor scores in chronic stroke patients.[13] In addition, real-time joint tracking with depth-sensing cameras makes motion analysis possible[14–16] and can provide quantitative results for movement quality, which is difficult using the manual assessment tool.[14, 15] Kinect is a relatively cheap sensor that needs no additional hardware, except for a computer, to acquire motion data for FMA.

The primary objectives of this study were to investigate whether Kinect motion data could be used to predict FMA score and whether predicted scores correlated with those measured by an experienced therapist in hemiplegic stroke patients. Secondarily, the usefulness of Kinect movement quality analysis was investigated.

## Materials and Methods

### Subjects

**Fig 1:**
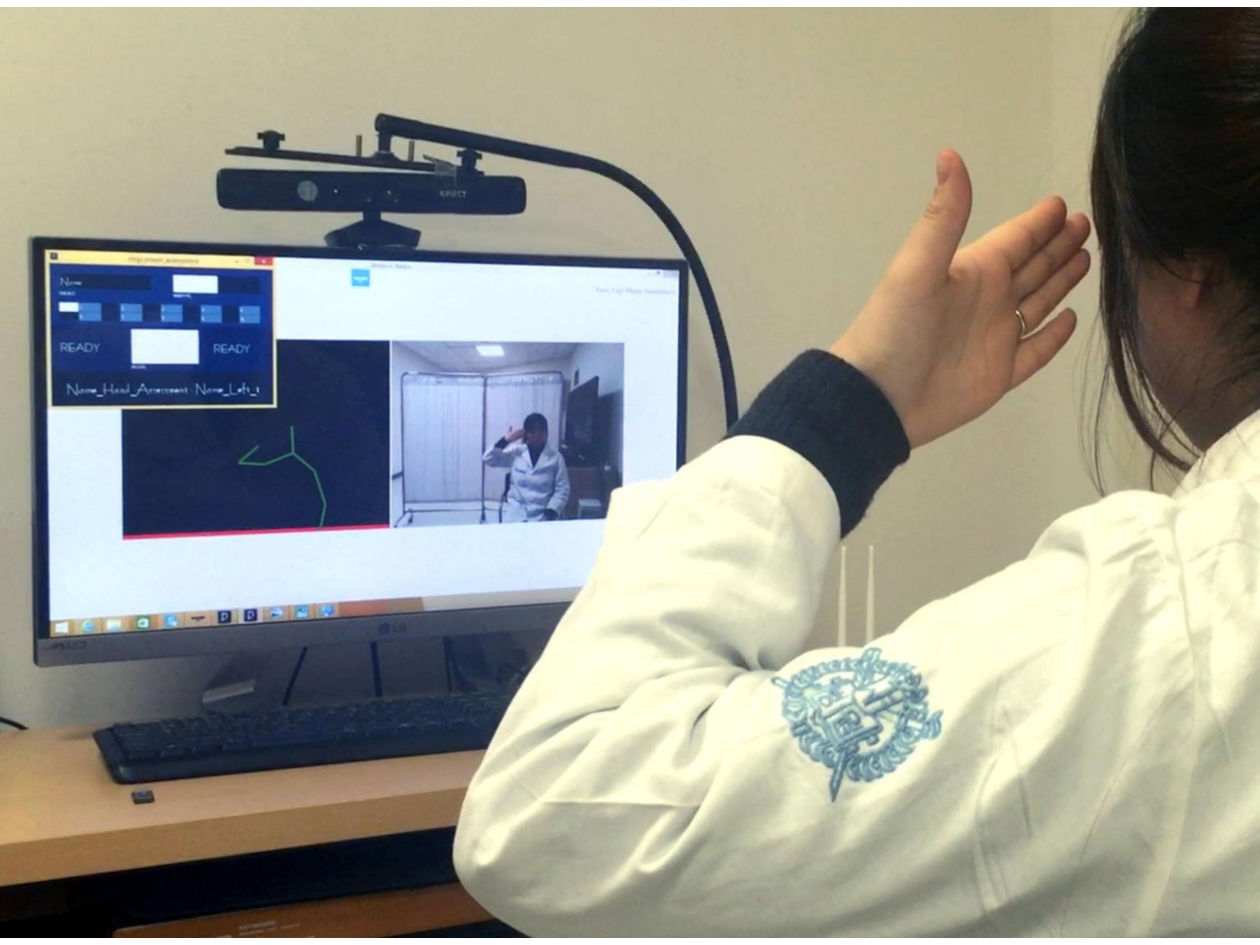
Motion data recording program. The recording program includes subjects' abbreviation, recording arm side, assessment item number. When pushing the record button and starting an item of Fugl-Meyer assessment, upper extremity skeleton of a subject can be shown in the monitor.

Patients were recruited from December 2013 to February 2015. Patients were eligible for inclusion if they had unilateral hemiplegia caused by ischemic or hemorrhagic stroke. Patients were excluded if they were younger than 18 years of age; had serious medical complications requiring intensive care, such as pneumonia, urinary tract infection, acute coronary syndrome, inability to provide written informed consent and any other conditions that might interfere with participation. All subjects received detailed information about the study and provided written consent. The individual in Fig 1 provided written informed consent (as outlined in the PLOS consent form) to publish the picture. This research protocol was approved by the Seoul National University Bundang Hospital institutional review board and was conducted in accordance with the regulatory standards of Good Clinical Practice and the Declaration of Helsinki (World Medical Association Declaration of Helsinki: Ethical Principles for Medical Research Involving Human Subjects, 2000).

### Experimental design

Subjects were seated comfortably in a chair to test UE FMA. Among the 33 items for UE evaluation, 13 were selected for Kinect motion data recording: flexor synergy (shoulder retraction, shoulder elevation, shoulder abduction, shoulder external rotation, elbow flexion, forearm supination), extensor synergy (shoulder adduction and internal rotation, elbow extension, forearm pronation), volitional motion mixing dynamic flexor and extensor synergy (hand to lumbar spine, shoulder flexion 0° to 90°) and volitional movement with little or no synergy dependence (shoulder abduction 0° to 90°, shoulder flexion 90° to 180°). One occupational therapist with two-year experience in the FMA test did the evaluations. Subject motion was recorded simultaneously by Kinect for all 13 items. Kinect motion data were saved as a separate file, which is upper-limb joint data including time. The saved data and FMA scores were transferred to an engineering department for analysis.

### System for recording motion data with Kinect

The Kinect depth-sensing camera was operated with a frame-rate of 30Hz and was positioned in front of each subject to track the entire arm during FMA motions. Before the motion was recorded, the therapist entered subject information including recording arm side and the recording assessment item number into the recoding program. The therapist activated the recording function using a recording button in the Graphical User Interface (GUI) panel (Fig 1) after instructing the subject concerning the assessment item to capture the subject’s response. Data were stored sequentially with time for the UE joint positions comprising 31 variables including time, and positions of the head, shoulder center, shoulder, elbow, wrist and hand. Data were saved in text file format.

### Data extraction and normalization of Kinect motion data

The recorded joint movement data from each FMA assessment were extracted. For the left arm, as an example, left hand, left wrist, left elbow, left shoulder, shoulder center and head joint position data were extracted. To match the coordinates of both arms for machine learning, the right side data was mirrored to the left side based on the sagittal plane of the subject. Then data from both arms could be put into the learning system together. Data recorded at the start and end of each motion were clipped by thresholding of the joint distance between frames. The detailed clipping process is described in the S1 Appendix. To remove the differences of seating locations and to normalize body size, all joint data were transformed by minus of initial shoulder center and by dividing the summation of each body length (i.e. wrist-elbow, elbow-shoulder and shoulder-shoulder center).

### FMA scoring based on pattern recognition from Kinect data

To predict a FMA score for each assessment item, an artificial neural network (ANN) among various pattern recognition algorithms was adopted. The prediction target of each item score (0, 1 or 2) was evaluated by one therapist. In machine learning and cognitive science, ANNs are statistical learning models inspired by biological neural networks that have become popular in solving various problems in diverse fields. In particular, it has been adopted to solve motion recognition problems in computer vision.[17, 18]

To properly classify motion patterns, features must be extracted from the captured motion data, which contains the positional information of every upper limb joint. Angles and distances between two joints (for example, hand-shoulder, hand-head and elbow-head) are computed from the original position data. Normalized jerky data based on jerky motion analysis is also used as an additional feature. In particular, bounding area and variance data for each feature are also used because the range of the motion increases as the FMA score increases.

The extracted features from motion captured data and the corresponding FMA scores that were evaluated by one therapist were used to train the ANN model. Predicting a score for each assessment depends on different features. Dimensionality reduction using principal component analysis (PCA) was performed to distinguish major features from all existing features. The original feature dimension was about 100 with slight variation from item to item. Reduction to between four and 10 dimensions was done for highly associated principle components. Therefore, different numbers of principal components were used to achieve the best accuracy for each assessment item. Dimensionality reduction is explained in more detail, in the S2 Appendix.

Thirteen assessments (26 scores in total) among all UE FMA determinations were predicted. An identical ANN structure (i.e. number of neurons, number of hidden layers and activation functions) was applied to predict a score for all assessments. However, a different number of principal components were selected for each assessment after PCA dimensionality reduction. Thus, the dimensions of input data depended on the assessments.

### Cross validation of FMA prediction models

Experimental data for each assessment were collected from 41 subjects. As both normal side and hemiplegic side data were collected for each subject, 82 motion data captures in total were used to train the ANN model. However, the collected score data displayed a skewed distribution for some assessments. Thus, it was not reliable for the validation to merely divide the collected data into training and testing data.

**Fig 2:**
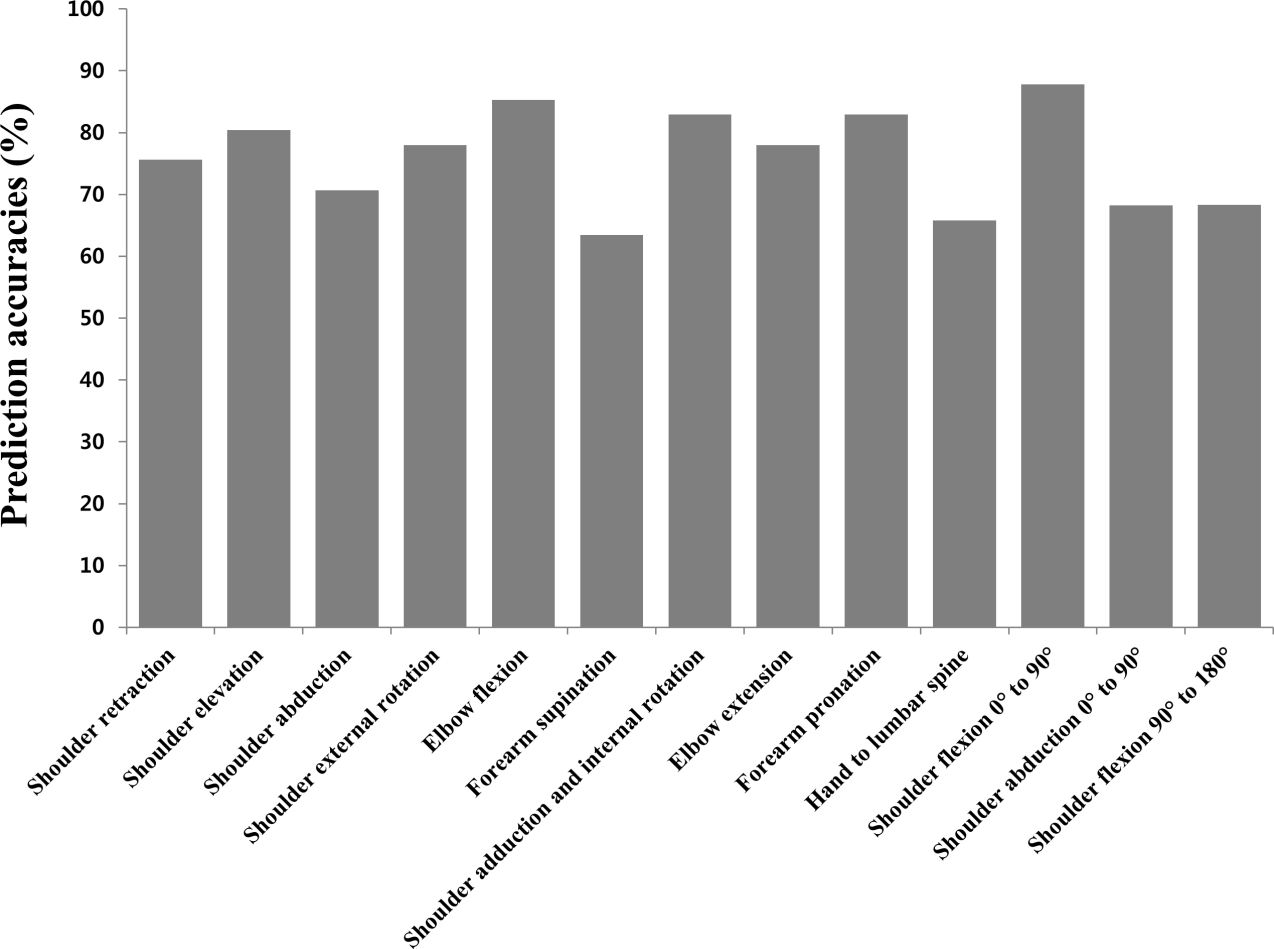
Prediction accuracies(%) of Fugl-Meyer assessment (FMA) scores using Kinect for real FMA scores in each item.

Using conventional validation, such as fixed partitioning the data set, the error of the training set is not a useful estimator of model performance and the error of the test data is not reliable in various testing data sets. Therefore, to reduce variability, multiple rounds of cross-validation were performed using different partitions. The validation results are averaged over the rounds and derive a more accurate estimate of model prediction performance. In this manner, 8- to 10-fold cross validations for each FMA item were performed. The k-fold means that the sample is randomly partitioned into k subsamples. One of the subsamples constitutes testing data and others are training data. Our cross-validation average error is shown in the prediction accuracy result (Fig 2). The overall process of this cross validation is described in more detail in the S3 Appendix.

### Assessing the degree of jerky motion using Kinect motion data

The evaluation of the movement impairment in this study is based on the integrated squared jerk.[19] The smoothness of motion is one element for subject assessment. The movement of the joint center was used for the Jerky motion analysis. An integrated jerky motion varies greatly with the duration and length (distance) of the movement.[20, 21] Because the movements of each patient have very different durations and lengths, comparison of each movement is difficult using only jerk data (third derivation of position data). In order to solve this issue, we generated integrated jerky motion data that were dimensionless.[19] Jerk is the third derivation of position data. The integration of the square of the jerk has a unit of duration^5^/length^2^. The value includes movement times and distance. Therefore, in order to remove the effects, we made the value dimensionless by multiplying the overall duration with the length. The dimensionless value therefore made the original data comparable. Because the integrated squared jerk varies with duration and size of the displacement, it was normalized by multiplying duration^5^/length^2^.[22] The squared root was taken to obtain a quantity proportional to the absolute jerk. The equation for normalized jerk is described in the following equation:

$$Jerk(t)=\frac{d^{3}\vec{P}}{dt^{3}}$$

$$(Normalized\;Jerk)=\sqrt{1/2\;\int_{T_{1}}^{T_{2}}{Jerk}^{2}(t)dt\times {duration}^{5}/{length}^{2}}$$

**Fig 3:**
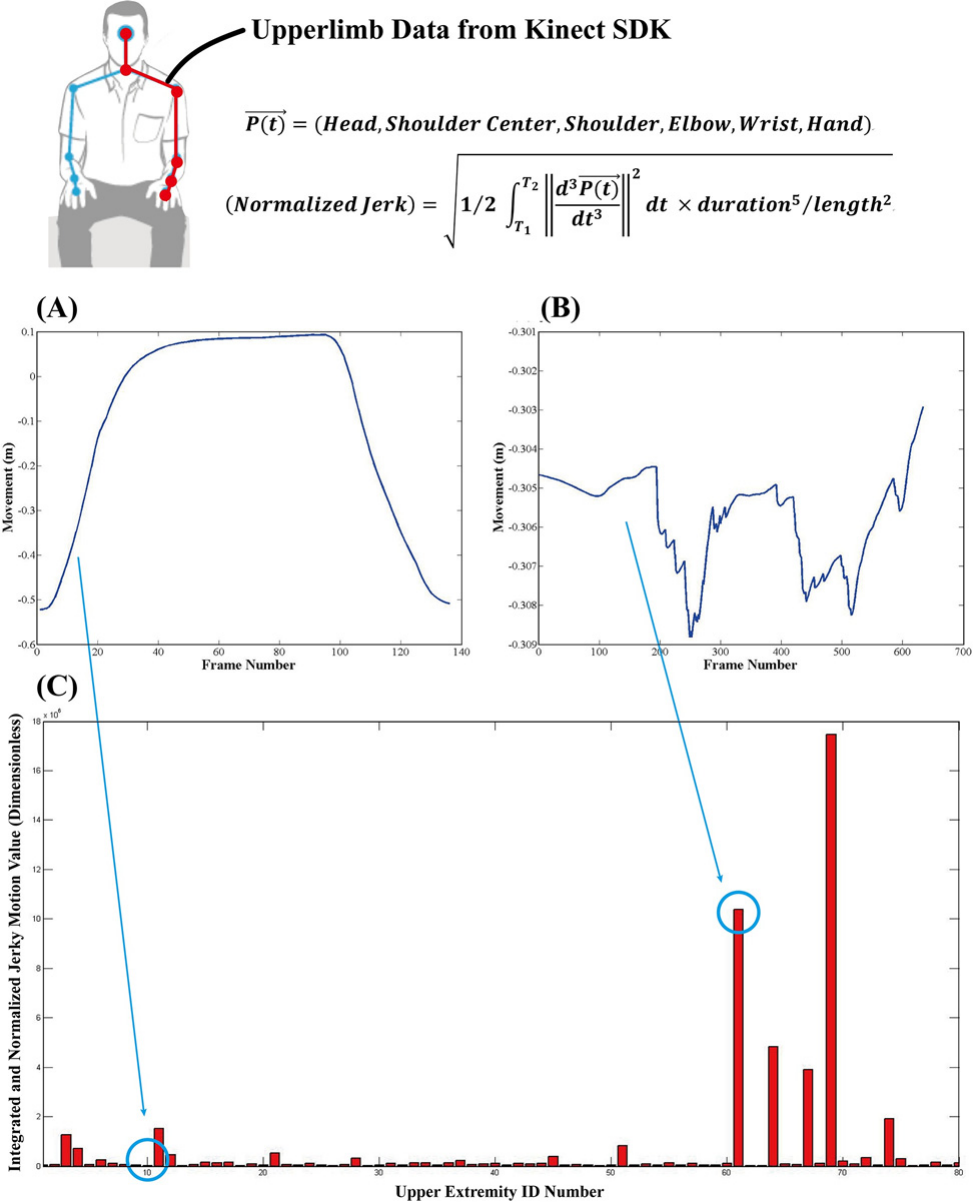
Jerky motion analysis. (A) and (B) are the results from y-direction hand movements from UE numbers 10 and 61 during the motion for the flexion synergy item in FMA. (C) is an example of the results of jerky motion analysis. A smooth curve movement like (A) has a lower jerky score value, whereas a high trembling curve like (B) has a higher jerky motion score.

$\vec{P}$ is a movement vector composed of the positions of the joint centers. Jerk(t) is an 18 dimensional vector because subject motion data has 18 variables (six joint x three dimension). Jerk^2^(t) is two-norm of the jerk vector. Duration is the length of the clipped data. Length is the maximum distance of a position vector (time *t*) from the initial (time *T*_1_), which is the greatest difference of motion distance from the start of motion. A higher jerky score derived from this method indicates more jerky movement (Fig 3). Jerky scores during the motion for flexion synergy in FMA were used for analysis.

### Statistical analyses

Continuous variables are presented as mean ± 1 SD for normally distributed data and as median with interquartile range for skewed data. Categorical variables are presented as frequencies (percentages). Prediction accuracies of FMA scores using Kinect for each item (number of subjects showing agreement in scores between FMA using Kinect and real FMA / 41 (total number of subjects)) were calculated and are represented as a percentage. It was regarded as accurate if FMA score using Kinect was exactly the same as real FMA for each item. The scores of the 13 items for FMA using Kinect and real FMA were summed to assess the correlation. Total score of FMA for selected items ranged from 0 to 26. Pearson’s correlation coefficients were calculated to see the correlation between FMA scores using Kinect and real FMA scores in the affected upper extremity for the selected items. In addition, correlation between FMA score using Kinect for selected items and total real FMA score (0–66 points) was investigated using Pearson’s correlation coefficient. Log (jerky scores) between hemiplegic and non-hemiplegic side were compared using paired t-test. Correlations between Brunnstrom arm stage[23] and log (jerky score) were investigated using Spearman’s correlation coefficient in subjects with Brunnstrom arm stage from 3 to 6. Statistical analysis was performed using SPSS version 18.0 (SPSS, Chicago, IL, USA). Data used for analysis are available from S1 File.

## Results

### Characteristics of the patients

Among 44 patients who agreed to participate, 41 completed the FMA. The other three patients were not removed from the study during the FMA but refused to do the test after enrollment. Demographic and clinical characteristics are summarized in Table 1.

**Table 1:** Baseline characteristics of patients (n = 41).

Variables	Results
Age, years^a^	62.6 (12.9)
Sex, no. (%)	
Male	29 (70.7)
Female	12 (29.3)
Time since onset of stroke, days^b^	21 (19)
Paretic side, no.(%)	
Right	15 (36.6)
Left	26 (63.4)
Type of stroke, no.(%)	
Ischemic	28 (68.3)
Hemorrhagic	13 (31.7)
Stroke lesion location	
Cortical	11 (26.8)
Subcortical	30 (73.2)
FMA score in the affected upper extremity^a^	42.5 (19.5)
NIH stroke scale^b^^[^^24^^]^	5 (3)
Brunnstrom stage (arm) ^b^	4 (3)
Brunnstrom stage (hand) ^b^	4 (4)

FMA-Fugl-Meyer Assessment, NIH-National Institute of Health.

^a^Mean (SD)

^b^Median (interquartile range)

### Prediction accuracies for FMA scores using Kinect for each item

Prediction accuracies of FMA scores using Kinect for real FMA in the hemiplegic side were above 70% in nine of the 13 selected items (Fig 2). Four items (forearm supination, hand to lumbar spine, shoulder abduction 0° to 90° and shoulder flexion 90° to 180°) showed prediction accuracies between 60% and 70%.

### Correlations between FMA scores using Kinect and real scores

**Fig 4:**
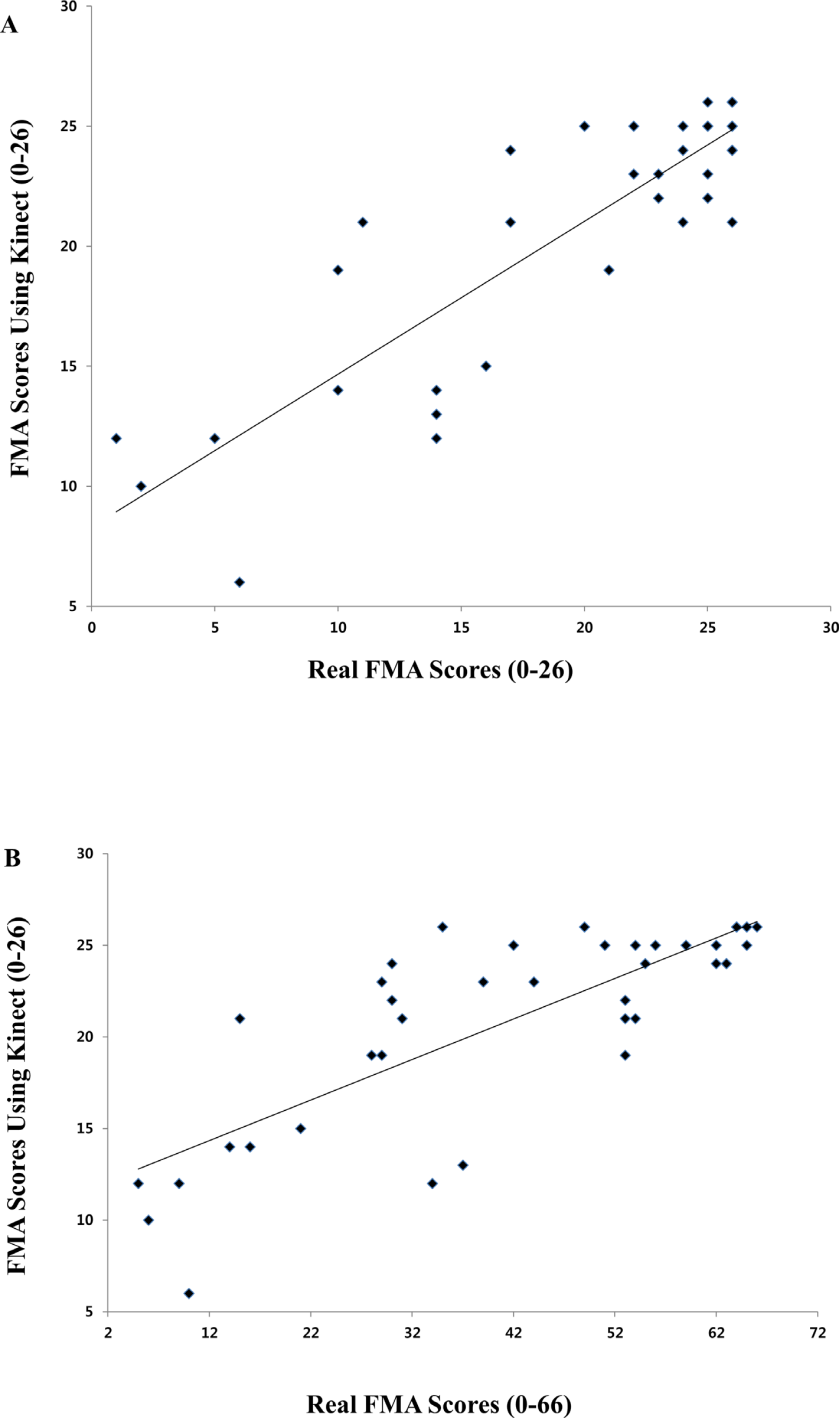
Correlation data. (A) Correlation between summed Fugl-Meyer assessment (FMA) using Kinect and real FMA scores for 13 selected items in the hemiplegic upper extremity (Pearson correlation coefficient = 0.873, *P*<0.0001). (B) Correlation between summed FMA using Kinect for the 13 items and real FMA scores for total of 33 items in the hemiplegic upper extremity (Pearson correlation coefficient = 0.799, *P*<0.0001).

Summed predicted FMA scores using Kinect for the 13 selected items showed high correlation with summed real FMA scores for 13 items in hemiplegic UEs (Pearson correlation coefficient = 0.873, *P*<0.0001) (Fig 4A). Correlation between summed predicted FMA scores using Kinect for the 13 selected items and summed FMA scores for the 33 items of the hemiplegic UEs were also high (Pearson correlation coefficient = 0.799, *P*<0.0001) (Fig 4B).

### Degree of jerky motion

**Fig 5:**
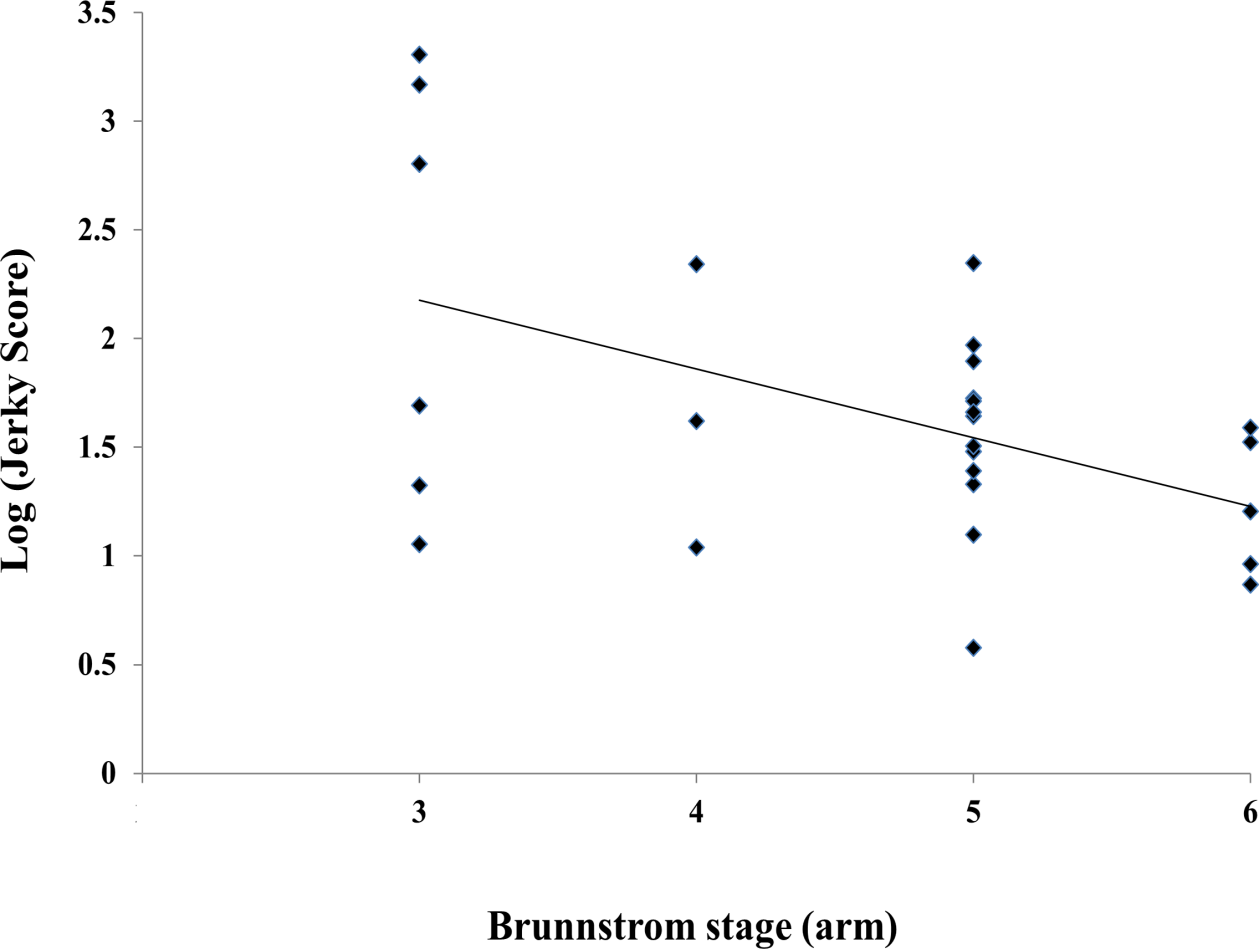
Correlation between log (jerky score) and Brunnstrom arm stage (3 to 6) in the hemiplegic upper extremity (n = 27) (Spearman correlation coefficient = -0.387, *P* = 0.046).

Jerky scores during flexion synergy motion (instructed to fully supinate the forearm, flex the elbow, and bring the hand to the ear on the opposite side) of FMA calculated from Kinect motion data were log transformed for normalization. Log (jerky score) in the hemiplegic UE was 1.81 ± 0.76 in the hemiplegic arm, which was significantly higher than that in the non-hemiplegic UE (1.21 ± 0.43) (P<0.0001). Log (jerky score) in the hemiplegic UE showed significant negative correlations with Brunnstrom arm stage (Spearman correlation coefficient = -0.387, P = 0.046) in the patients with Brunnstrom arm stage from 3 to 6 (n = 27) (Fig 5).

## Discussion

In this study with hemiplegic stroke patients, predicted FMA scores using Kinect were highly correlated with real FMA scores. In addition, jerky scores calculated from Kinect motion data can assess the degree of motion smoothness quantitatively, which can not be provided by conventional FMA with observations.

A few tools in the virtual environment to assess the motor impairment after stroke have been investigated in several prior studies, but the correlations with conventional assessment tools were modest (correlation coefficient: 0.53–0.66).[8, 9, 25] One study evaluated the activities of daily living (ADL) in the virtual environment and compared it with the scores of Wolf Motor Function Test (WMFT).[25] Because WMFT is assessed during actual manipulation of objects, recognition of object's size, weight and texture and sensory feedback is important but the virtual ADL assessment tool using Kinect cannot give this haptic feedback.[25] This limitation may be associated with a modest correlation; an additional device and expense will be required to overcome this limitation. Although two studies using manipulating devices with virtual realities showed modest correlations with conventional assessment tool, additional costs and space are required.[8, 9] In this context, FMA is the best assessment tool which can be predicted using motion tracking with Kinect. Kinect is a relatively inexpensive depth-sensing camera and no additional space and devices are required. The purchase cost continues to decrease and camera performance continues to increase. FMA scoring using Kinect has potential as valid assessment tool for motor function after stroke in the home-based rehabilitation setting.

FMA is valid and is widely used for motor function assessment in stroke patients. But, many items place a time burden on the assessor and patients. In this context, there have been efforts to reduce the FMA item; one study suggested reducing the items for UE evaluation to six.[26] Because of the limitation of Kinect to track various UE motions, only 13 FMA items were presently included. The correlation was high between summed FMA scores using Kinect and real FMA scores for the 13 selected items. Although correlation between summed FMA scores using Kinect and real FMA scores for all 33 UE hemiplegic items was reduced, the correlation coefficient of 0.799 was still high. This indicates that the number of items used in FMA using Kinect could feasibly be decreased, which would decrease the burden on patients and caregivers during assessment.

Coordinated movement is impaired after stroke; motions are not smooth but rather become jerky. Traditional Brunnstrom recovery phase reveals the recovery of coordinated movement and emergence from synergistic movements.[23] Quantitative measurement of movement smoothness using a robotic device has revealed improvement during the recovery after stroke.[27] Jerky scores using Kinect in our study were well negatively correlated with Brunnstrom stage. This quantitative measure can be used for follow-up of changes in movement in a manner that equivalent in quality to robotic devices but less expensive.

The FMA scoring system using only one Kinect in this study does have some limitations. One is the occlusion of the body part during tracking by Kinect. For instance, Kinect can not track the hand when it is moved to the lumbar spine for FMA. One of the solutions for the occlusion problem is using multiple Kinect Sensors, but this may be associated with increased cost. Wearable sensors such as smart watches or wrist bands providing positional information can also be applied to solve the occlusion problem in our system. Another limitation is that the Kinect Software Development Kit is not appropriate for hand tracking of FMA motion, because it is unable to track pronation/supination, radial/ulnar direction and hand grasp below the wrist during full-body tracking. Some other FMA scores that could not be predicted in this research are required to detect the above hand information with accurate hand position tracking. The items in FMA showing low prediction accuracy are forearm supination, shoulder abduction 0° to 90° and shoulder flexion 90° to 180°, which require the information on forearm rotation. If the resolution for the hand during full-body tracking with Kinect is increased in the future, the prediction accuracy for items including information on forearm rotation are expected to improve and the FMA items excluded in this study can be added. While waiting for a more advanced form of Kinect, another solution is fusion with other hand tracking sensor, such as the Leap Motion device (Leap Motion, USA), which allows precise hand tracking using a hand point cloud below the wrist. We plan to use Leap Motion for precise hand tracking, and add more items of UE FMA.

More and varied movement data of each assessment item would increase the precision of the FMA system score using Kinect. Imbalance of real FMA scores decreases the prediction accuracies of each item. Techniques explored to solve the imbalance issues include re-sample techniques,[28] adaptive training algorithms,[29] particle swarm optimization[30] and parameter searching. We used re-sample techniques and cross-validation to minimize errors from dataset imbalance. However, the best way to solve this problem is to gather the more patient data. Although we used the data from 41 stroke patients with various motor impairments, decrease of the imbalance by collecting more data sets and adoption of the above techniques could help increase prediction accuracies. In further work, web-based uploading system of FMA Kinect motion data and real FMA data in various area could help acquire more patient data. Furthermore, use of a cloud computing system with machine learning ability, such as Microsoft Azure ML, Amazon Machine Learning or IBM Watson Analytics, will facilitate develop of a prediction model capable of self-learning whenever new patient data is uploaded, and to predict FMA score using the model in the absence of a specialist. In our study, the recording of FMA using Kinect was conducted in the hospital with the supervision of a therapist. Further study to validate the usefulness of advanced system in the real home-setting is required.

## Conclusions

The FMA scoring system using Kinect is valid and provides additional quantitative measures of motion smoothness in stroke patients. This tool has the potential to be a useful and inexpensive tele-assessment tool of post-stroke motor function in the home-based setting. Acquisition of more patient data may increase the accuracy of this tool. Further study to validate this tool in the home-based setting is required.

## Supporting Information

S1 Appendix Data clipping. (DOCX)

S2 Appendix Dimensionality reduction. (DOCX)

S3 Appendix Overall process of FMA prediction. (DOCX)

S1 File Data file used for analysis. (XLSX)
