# Supplementary material for: Upper Extremity Functional Evaluation by Fugl-Meyer Assessment Scoring Using Depth-Sensing Camera in Hemiplegic Stroke Patients
Source: PLoS One. 2016 Jul 1;11(7):e0158640. doi: 10.1371/journal.pone.0158640 (PMC4930182; doi:10.1371/journal.pone.0158640)
Supplement: S1 Appendix — (DOCX) [file pone.0158640.s001.docx]

**Data clipping**

Data clipping is an important process because the intervals from the start of recording to the start of movement as well as the intervals from the end of end to the end of recording vary between each recording and each subject. So, we needed to clip the interval from the start to the end of the movement in each recording for analysis. We converted the joint position in each frame to eighteen dimension vectors (3 axis x 6 joints), and used the velocity vectors for differentiation. We calculated the magnitude (2-norm) of the velocity vector in each frame (average of velocity divided by average magnitude in all frames). While looping the frames forward from the first frame, the starting point was determined when the magnitude of the velocity vector was higher than the mean magnitude. While looping the frames backward from the last frame, the end point was determined when the magnitude of the velocity vector was higher than the mean magnitude. An example of the data before and after clipping is presented in Fig 1.


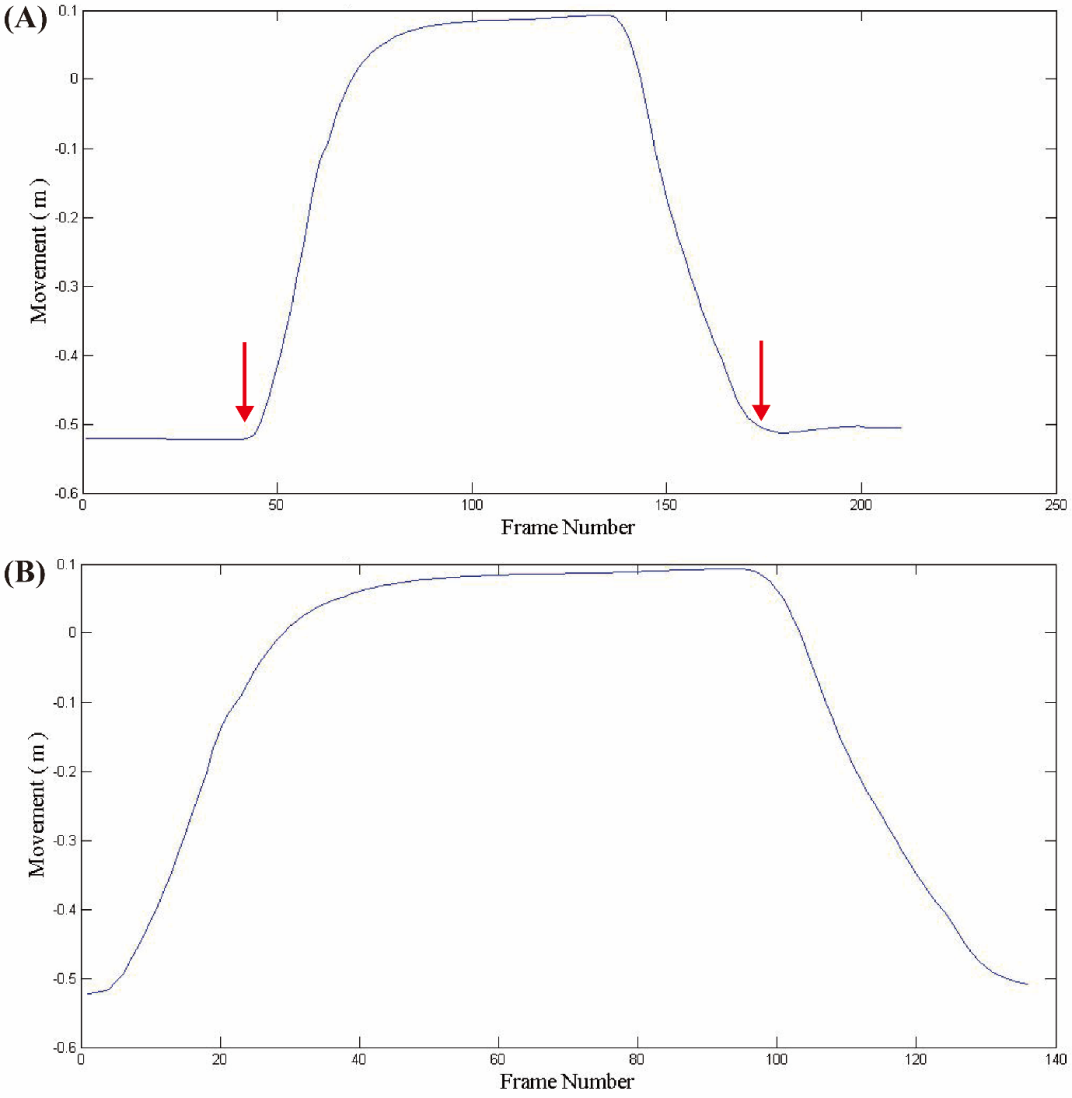


Fig 1. Data before and after clipping. (A) Time series data for y-Direction movement of hand center before clipping process. Red arrows are clipping points. (B) Time series data for y-Direction movement of hand center after clipping process.
