# Supplementary material for: Upper Extremity Functional Evaluation by Fugl-Meyer Assessment Scoring Using Depth-Sensing Camera in Hemiplegic Stroke Patients
Source: PLoS One. 2016 Jul 1;11(7):e0158640. doi: 10.1371/journal.pone.0158640 (PMC4930182; doi:10.1371/journal.pone.0158640)
Supplement: S2 Appendix — (DOCX) [file pone.0158640.s002.docx]

**Dimensionality reduction**

PCA transforms the data into a new coordinate system with greater order of variance based on the projection of the data. We considered that smaller variance dimensions might be not related to FMA score, which means that not all the principal components need to be retained for learning. So, we used the first to the ‘n’th components. For instance, we used the first and second components for further machine learning in some cases, presented in Fig 1. Different numbers of principal components were used to achieve the best accuracy for each assessment item.


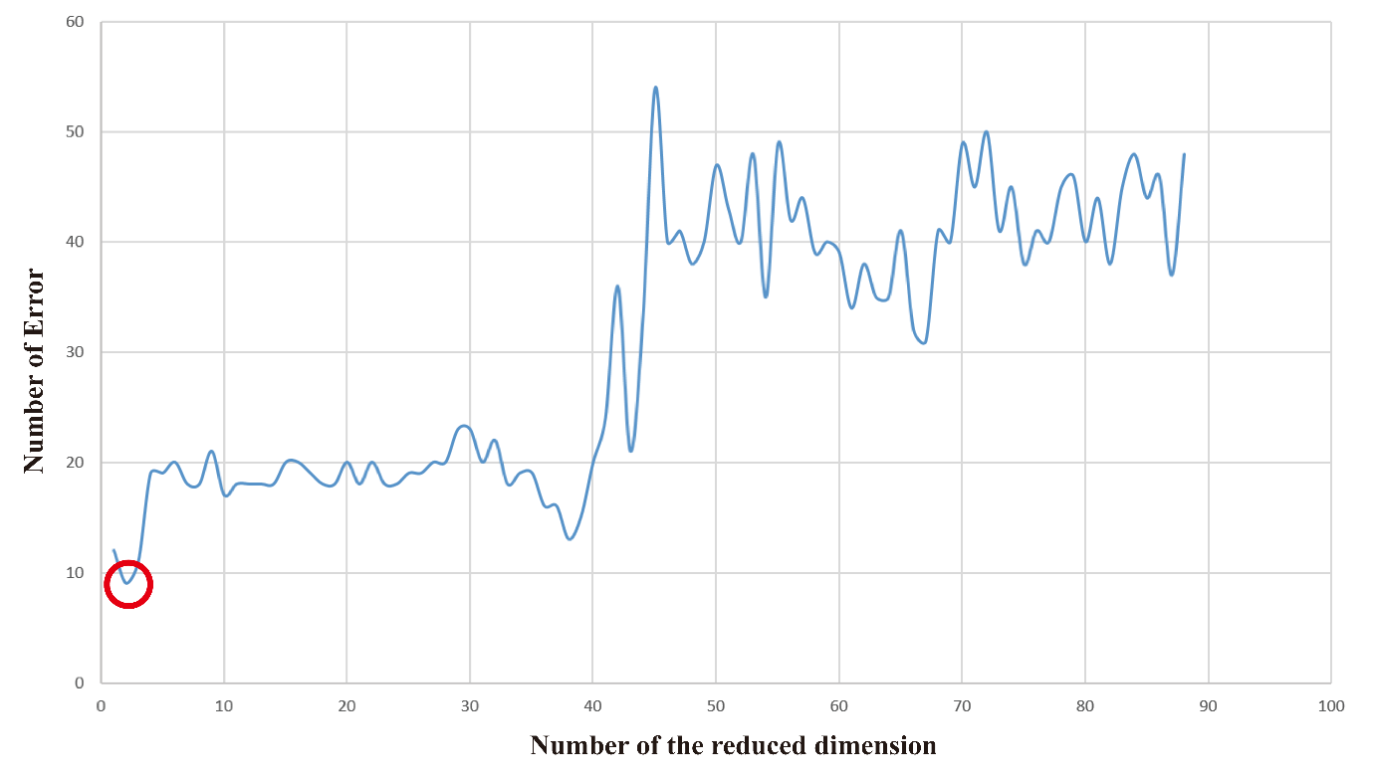
**Fig 1. Dimensionality Reduction.** The number of false prediction errors with the use of various numbers of principle components in the flexor synergy movement.
