# Supplementary material for: Upper Extremity Functional Evaluation by Fugl-Meyer Assessment Scoring Using Depth-Sensing Camera in Hemiplegic Stroke Patients
Source: PLoS One. 2016 Jul 1;11(7):e0158640. doi: 10.1371/journal.pone.0158640 (PMC4930182; doi:10.1371/journal.pone.0158640)
Supplement: S3 Appendix — (DOCX) [file pone.0158640.s003.docx]

**Overall Process of FMA prediction**

The overall process and input/output is shown in Fig 1. The original input is data recorded by Kinect with 18 dimensions in each frame, but the number of frames varies between subjects. Before dimension reduction using principle component analysis (PCA), we have a large element feature vector including the position, angle and distance. In the artificial neural network, the number of inputs for the neural network is different in the FMA item because we used PCA dimension reduction. The input is dimension reduced data, and the output is one of three levels (0, 1, or 2), because each FMA item has three level scores (0, 1 and 2).


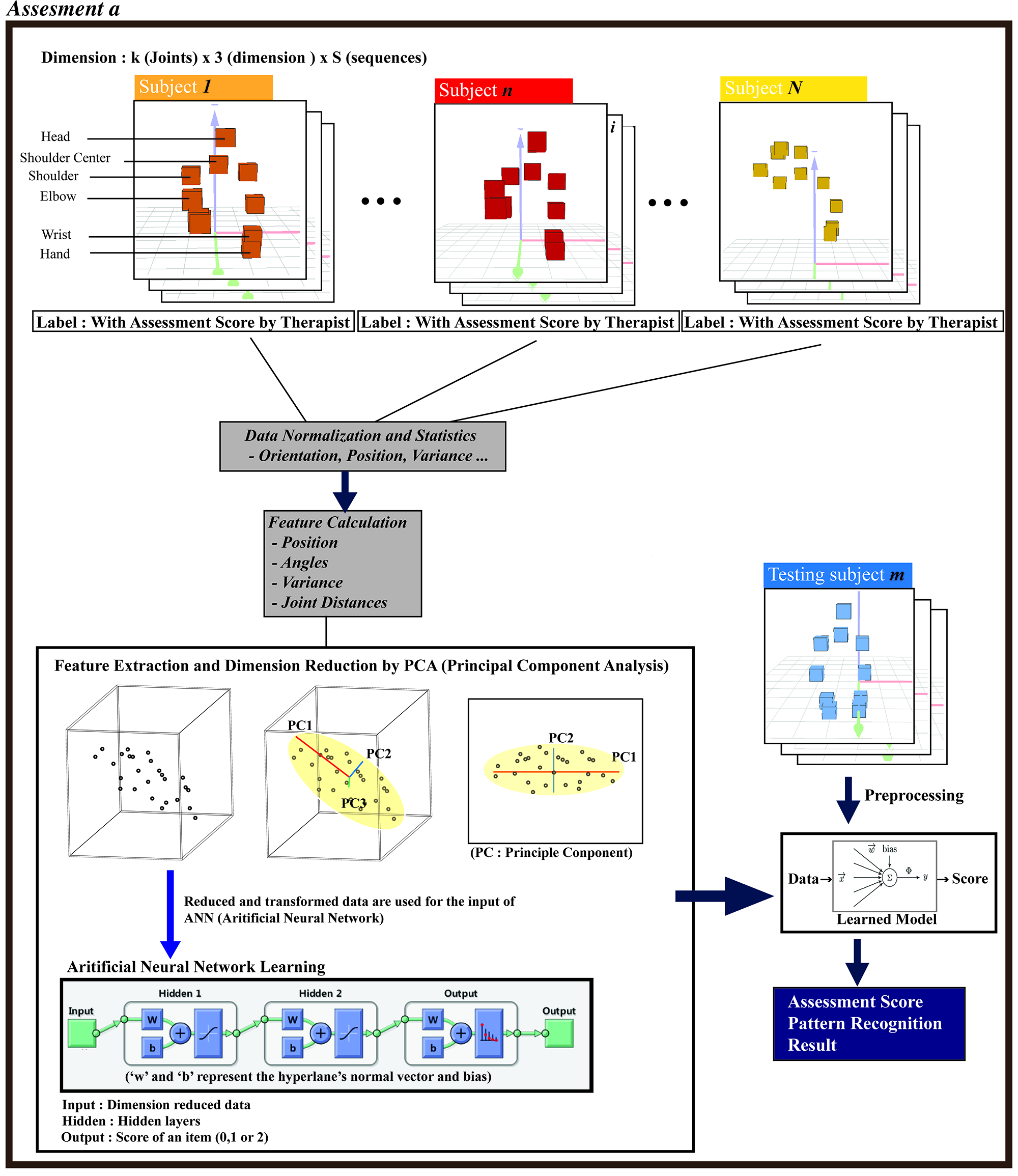


Fig 1. Overall process of our FMA prediction. All recorded position data with their label (FMA item score) was transformed to feature vector with same dimension. After principal component analysis dimension reduction process, the transformed data was used to find models of artificial neural network (ANN)
